# Laboratory Tests on the Possibility of Using Flax Fibers as a Plant-Origin Reinforcement Component in Composite Friction Materials for Vehicle Braking Systems

**DOI:** 10.3390/ma17122861

**Published:** 2024-06-12

**Authors:** Andrzej Borawski, Dariusz Szpica, Grzegorz Mieczkowski

**Affiliations:** Faculty of Mechanical Engineering, Bialystok University of Technology, 45C Wiejska Str., 15-351 Bialystok, Poland; a.borawski@pb.edu.pl (A.B.); g.mieczkowski@pb.edu.pl (G.M.)

**Keywords:** brakes, friction, wear, tribology, flax fibers, aramid fibers, statistical methods

## Abstract

Braking systems are extremely important in any vehicle. They convert the kinetic energy of motion into thermal energy that is dissipated into the atmosphere. Different vehicle groups have different nominal and maximum speeds and masses, so the amount of thermal energy that needs to be absorbed by the friction pads and then dissipated can vary significantly. Conventional friction materials are composite materials capable of withstanding high temperatures (in the order of 500–600 °C) and high mechanical loads resulting from braking intensity and vehicle weight. In small vehicles traveling at low speeds, where both the amount of thermal energy and its density are limited, the use of slightly weaker friction materials with better ecological properties can be considered. This work proposes a prototype composite friction material using flax fibers as reinforcement instead of the commonly used aramid. A number of samples were prepared and subjected to laboratory tests. The samples were prepared using components of plant origin, specifically flax fibers. This component acted as reinforcement in the composite friction material, replacing aramid commonly used for this purpose. The main tribological characteristics were determined, such as the values of the coefficients of friction and the coefficients of abrasive wear rate. For this purpose, an authorial method using ball-cratering contact was used. The results were analyzed using statistical methods. It was found that the composite material using flax fibers does not differ significantly in its tribological properties from conventional solutions; so, it can be assumed that it can be used in the vehicle’s braking system.

## 1. Introduction

Friction is one of the most common physical phenomena encountered by mankind. It is used by many industries, including the automotive industry. In the automotive industry, the most important application of friction is in braking systems [1,2].

The vast majority of vehicles use a solution where a rotating brake disc works with stationary pads that slide on either side. It is, therefore, a kind of motor that converts the kinetic energy of motion into thermal energy that is then dissipated into the environment [3].

The amount of heat energy produced depends on many factors [4]. Current development trends in environmental protection suggest that the weight of the vehicle should be as low as possible. This has a positive effect not only on braking but also on the vehicle’s performance (especially acceleration). It also reduces rolling resistance, which has a positive effect on fuel consumption. The second factor, vehicle speed, is strictly dependent on the speed limit on a given stretch of road. These, in turn, are on a downward trend, and drivers who fail to comply with the limits are subject to increasingly severe penalties, which can even include confiscation of the vehicle or imprisonment. This is especially important in urban areas, where the speed limit is usually 50 km/h. Such a rule has a definite positive impact on both safety (stopping distance is reduced) and the life of the braking system due to lower thermal and mechanical loads [5,6].

The number of vehicles on the earth continues to grow. It is estimated that by 2030, there will be 2 billion vehicles on the planet. Each of these vehicles is equipped with friction materials that wear out and these wear products are released into the atmosphere. It is, therefore, important that the production, operation, and disposal (recycling) processes are as minimally invasive and toxic as possible. Numerous tests are carried out on materials that may be used in the working elements of braking systems. Products of natural origin (metals, minerals, plants or animals) or of artificial origin (plastics, ceramics, etc.) are used. All these components can be classified according to their function. Based on this criterion, there are binders, reinforcements, fillers, and abrasives [7,8]. The list of currently used components contains several thousand examples. Each manufacturer selects about 10–20 of them, measures the appropriate proportions, and then creates a final product with specific properties.

The component that significantly influences the properties (especially mechanical) of the friction material is its reinforcement. Asbestos worked very well in this role, but its carcinogenic properties [9] meant that it was completely withdrawn from the automotive industry. Much research has since been carried out to develop a substitute. The most commonly used reinforcements today are synthetic materials such as carbon fiber and aramid [10,11]. Less often, more exotic materials of natural origin are used. These could be palm kernel shells [12], banana peels [13], periwinkle shells [14], or cocoa bean shells [15]. What is important is that they are obtained as a by-product of other processes (such as food production). Unfortunately, they have poor mechanical properties; this characteristic limits their use as reinforcements in brake pads.

On the other hand, synthetic materials have good mechanical properties. However, their production process often involves hazardous chemicals [16].

Today, aramid fibers are produced via the low-temperature polycondensation of para-phenylenediamine (PPD) and terephthaloyl chloride (TCL) monomers. PPD is a highly sensitizing aromatic amine used in hair, fur, and fabric dyeing. The by-product is hydrochloric acid. The production of aramid fibers and fabrics is expensive due to the difficulty of using concentrated sulfuric acid, which is required to maintain the water-insoluble polymer during synthesis and spinning [17,18].

Another harmful material often used as a reinforcement in friction materials is carbon fiber. Their production and processing raise three issues: dust inhalation, skin irritation, and the effect of the fibers on electrical equipment [19,20].

These problems make it necessary to look for a more ecological solution. As shown in previous work [21], the use of materials of plant origin as reinforcement is promising, especially for use in light vehicles moving at low speeds (e.g., urban traffic). However, the materials used so far [12,13,14,15] usually caused serious changes in the properties of the materials. Moreover, none of them were used as reinforcement. In this work, we decided to investigate how different variations in the concentration of alternative reinforcement in the form of flax fibers, replacing aramid fibers, would affect the tribological properties of the composite friction material.

## 2. Materials and Methods

A number of samples with different compositions were produced. The basic group of samples was S1, whose composition was similar to that of commercial brake pads. In the other groups (S2…S4), the conventional aramid reinforcement was gradually replaced by flax fibers (Table 1). The main differences between the groups are as follows:

- S1: Aramid reinforcement only—reference sample;
- S2: Approximately 33% of aramid was replaced with flax fibers;
- S3: Approximately 66% of aramid was replaced with flax fibers;
- S4: Only flax reinforcement was used, completely eliminating aramid.

**Table 1:** Composition of individual groups of samples.

Component	Contents, wt. %			
	S1	S2	S3	S4
Brass powder, diameter < 0.1 mm (CuZn20)	12	12	12	12
Cooper powder, diameter < 0.2 mm (Cu)	25	25	25	25
Steel chips, 0.5 < length < 5 mm (0.18% C, 0.5% Si, 1.65% Mn, 0.05% P, 0.02% S, 0.08% Mo)	7	7	7	7
Aramid fibers, 3 < length < 5 mm	12	8	4	0
Flax fibers, 3 < length < 5 mm	0	4	8	12
Resin	17	17	17	17
Graphite powder, diameter < 0.5 mm (C)	5	5	5	5
Fly ash powder, diameter < 0.2 mm	18	18	18	18
Cast iron chips, 0.5 < length < 5 mm EN-GJS-400-12	4	4	4	4

**Figure 1:**
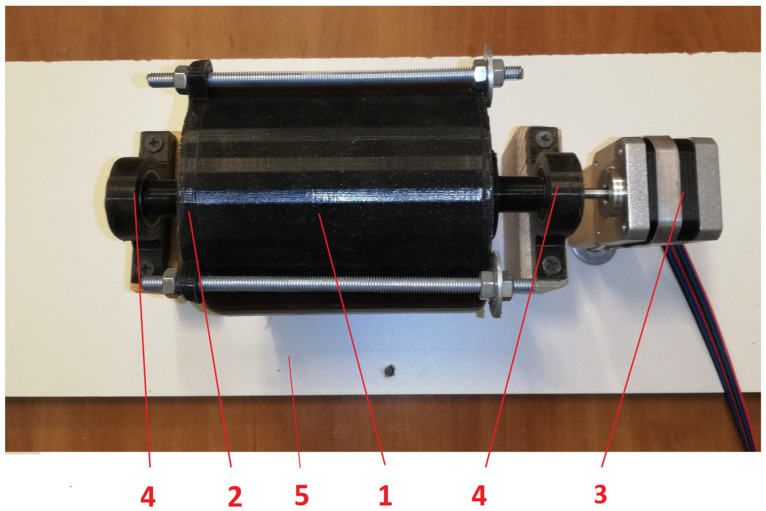
Three-dimensional printed mixer used in material preparation process: 1—container with mixing blades inside; 2—cover; 3—steeper motor (for precise rotation controlling); 4—bearing; 5—base.

The components were measured using a Steinberg SBS-LW-300A (Steinberg, Hamburg, Germany) precision balance (accuracy 10^−3^ g). The prepared mixture was placed in a mixing device (Figure 1), which was built using additive technology. The device was driven by a Nema 17 (Changzhou, China) stepper motor connected to an Arduino (Arduino S.r.l., Monza, Italy) controller so both the mixing time and the mixing parameters were arbitrary. In the present tests, the mixing speed was set at 3 rpm, and the mixing time was two hours.

**Figure 2:**
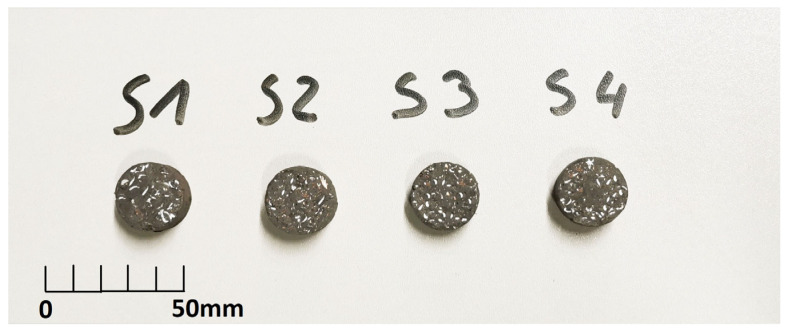
Selected research samples from individual groups.

Once the mixture was ready, it was placed in molds and then subjected to a pressure of 20 MPa. This was achieved by using a hydraulic press. The samples were left there for 12 h. They were then removed from the molds and heated at 60 °C for 24 h. Finally, the tested surfaces were shaped using a grinder. This produced a flat surface with a ninth-degree roughness. Examples of samples that were finally shaped into cylinders 1″ in diameter and 10 mm thick are shown in Figure 2.

**Figure 3:**
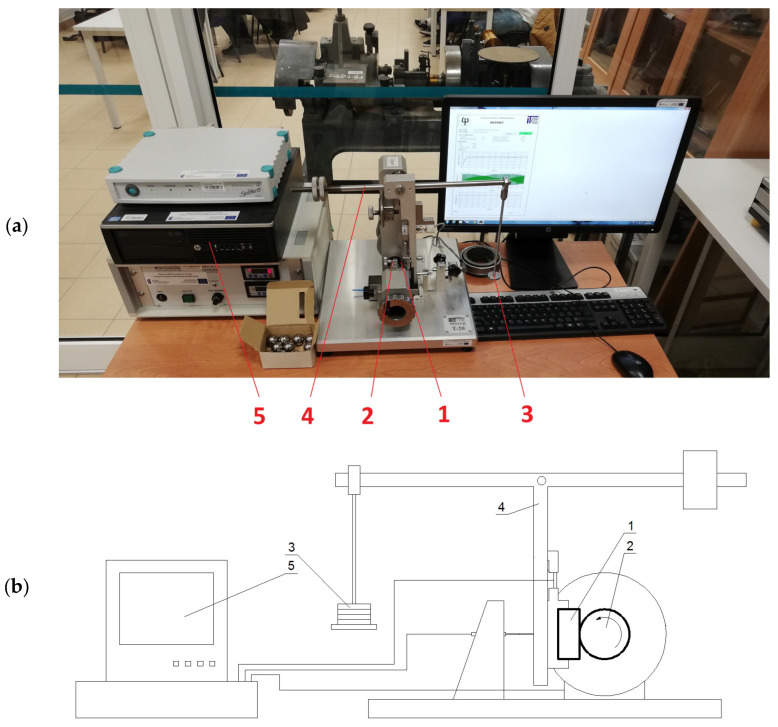
T-20 test stand: (**a**) picture; (**b**) sketch. 1—sample; 2—sphere (counter-sample); 3—loaded mass; 4—three-arm leaver; 5—computer.

All tests were carried out using the author’s method described in detail in [22]. This is a ball crater contact method. The laboratory station used was T-20 (Figure 3). In this station, the sample (1) is placed in the holder of the lower lever arm (4). The mass (3) loaded on the second arm of the lever (4) causes the lever to rotate, pressing the sample (1) against the rotating counter sample (2), which, in this case, is a cast iron (EN-GJS-400-12, hardness approx. 180 HB) ball with a diameter of 25.4 × 10^−3^ m, made with a roundness tolerance of less than 0.001 mm. The test parameters, i.e., friction force, displacement (ball depth), and, after connecting the sensor, temperature, are recorded by the connected computer (5). At the end of the tests, the length of which can be determined by the number of revolutions or the test time, the software generates a report of the measurement process. The immediate result of the tests is the time profile of the friction force value and the craters created in the sample.

In this method, it is very important to design the experiment correctly, where the key is the choice of the input parameters (ball speed, total friction path, and compression force). Among the many design methods analyzed, a method based on the experimental optimization of the quality of multi-parameter processes (Taguchi method [23,24]) was selected. It focuses primarily on the consequences of quality loss, not its increase. It is assumed that each process parameter generates a loss that is inversely proportional to the quality. Therefore, the concept of the loss function, the quality of the *η* coefficient—the ratio of the signal *S* to the noise *N*—and the orthogonal tables [25] are of fundamental importance. In this case, since we have three input parameters, the orthogonal array takes the form shown in Table 2.

There are nine verses in this table. Each of them contains a unique combination of input parameters for preliminary experiments. Tests performed in this configuration gave the result shown in Table 3.

**Figure 4:**
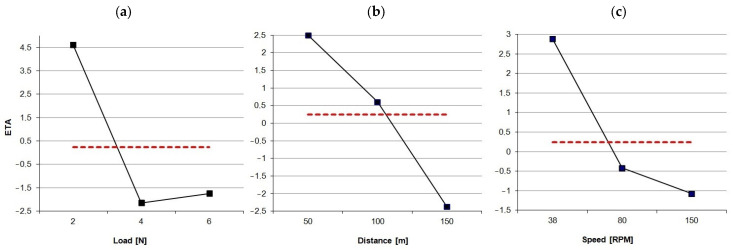
ETA function diagram for each parameter: (**a**) load; (**b**) distance; (**c**) speed; red dot line—average ETA value.

Then, we use the criterion “the bigger the better”, described by the following formula:

(1) $$\eta =-10{log}_{10}\left(\frac{1}{n}\sum_{i=1}^{n}y_{i}^{2}\right),$$

where: *η* (ETA)—signal-to-noise ratio (S/N) function; *n*—number of measurements for a single sample (repeated five times in this case); *y*—average friction force value of a single test allowed for the designation of functions (Figure 4). This, in turn, made it possible to finally determine the input parameters of the main experiment, respectively:

- Load: *L* = 2 N;
- Distance: *S* = 50 m;
- Rotation speed: *n* = 38 RPM.

As mentioned above, experiments create a crater in the sample. Its shape is a section of a sphere, and the diameter of the crater is strictly dependent on the amount of wear. After measuring in two planes (in line with and perpendicular to the friction track), taking the arithmetic mean, and inserting it into Archard’s formula [26,27], i.e.,

(2) $$k_{c}=\pi \frac{b^{4}}{64RSL},$$

where: *R*—the counter-sample radius; and *b*—arithmetic average of the measurements of the crater diameters (for this purpose the Delta Optical microscope and Brinell magnifying glass were used), it is possible to determine the abrasive wear rate (*k_c_*).

## 3. Results

**Figure 5:**
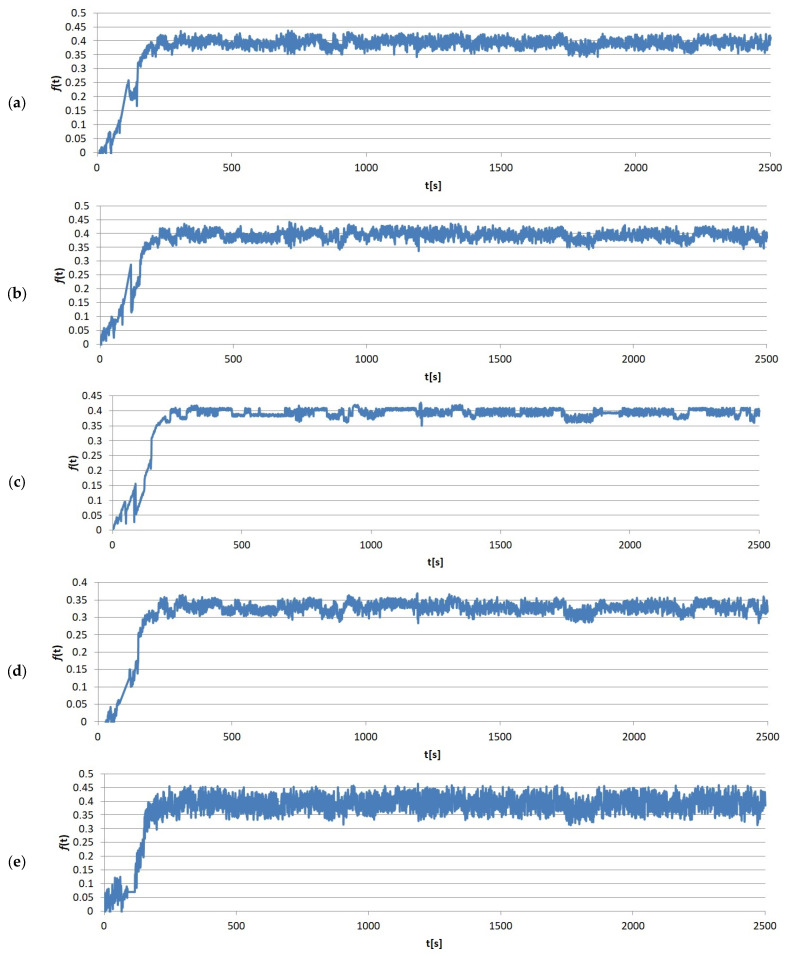
Example results graphs of the coefficient of friction of sample no. 1 from group S1: (**a**) run 1; (**b**) run 2; (**c**) run 3; (**d**) run 4; (**e**) run 5.

**Figure 6:**
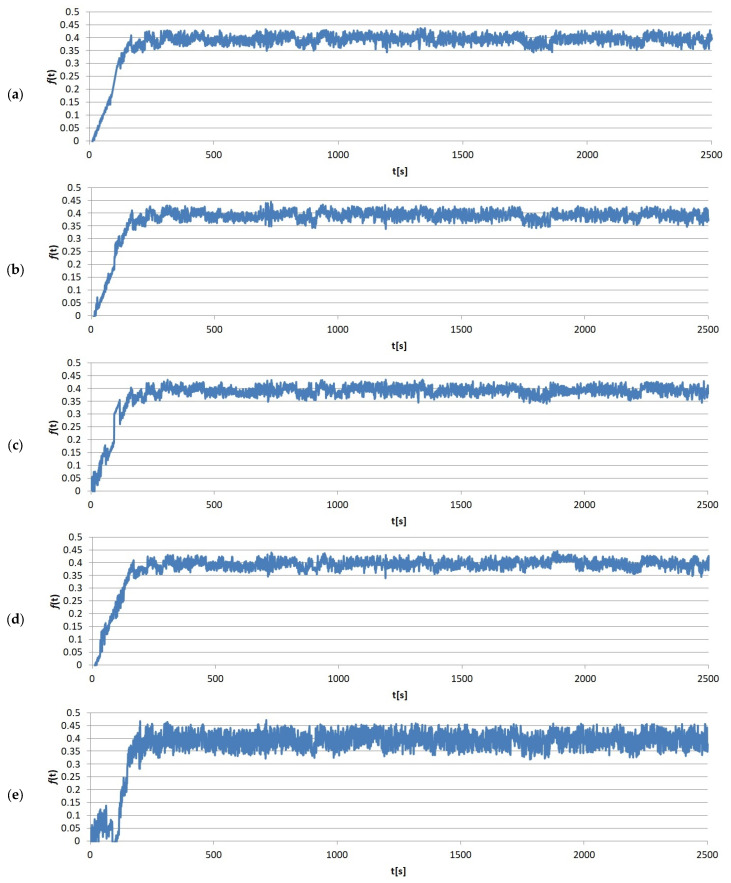
Example results graphs of the coefficient of friction of sample no. 4 from group S2: (**a**) run 1; (**b**) run 2; (**c**) run 3; (**d**) run 4; (**e**) run 5.

**Figure 7:**
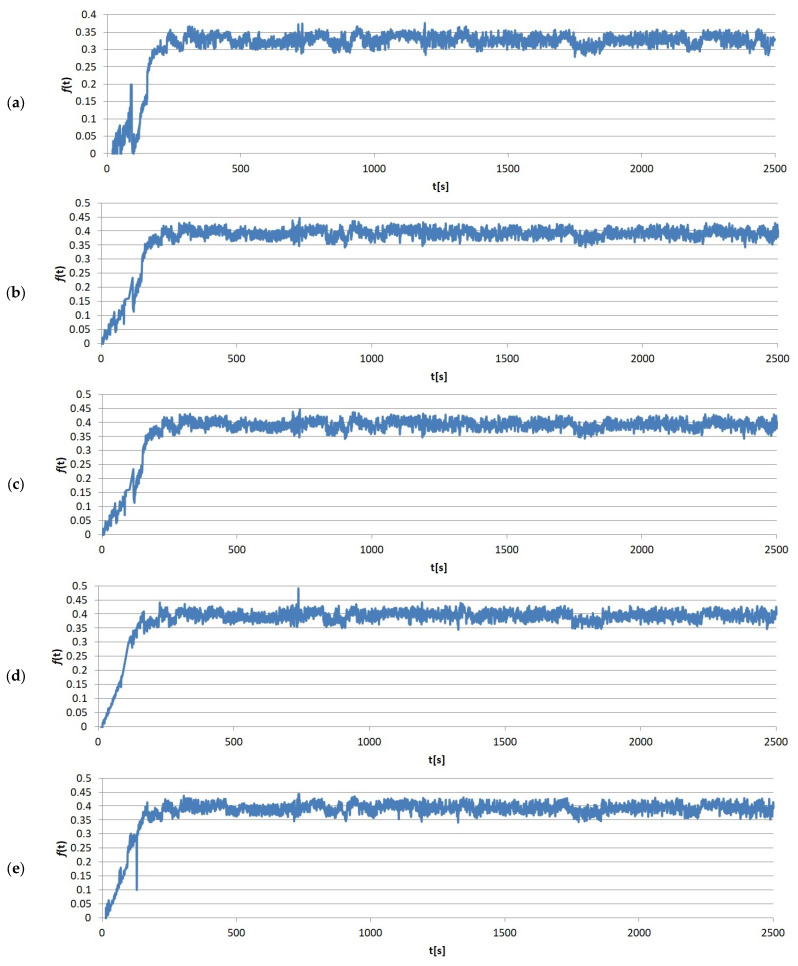
Example results graphs of the coefficient of friction of sample no. 7 from group S3: (**a**) run 1; (**b**) run 2; (**c**) run 3; (**d**) run 4; (**e**) run 5.

**Figure 8:**
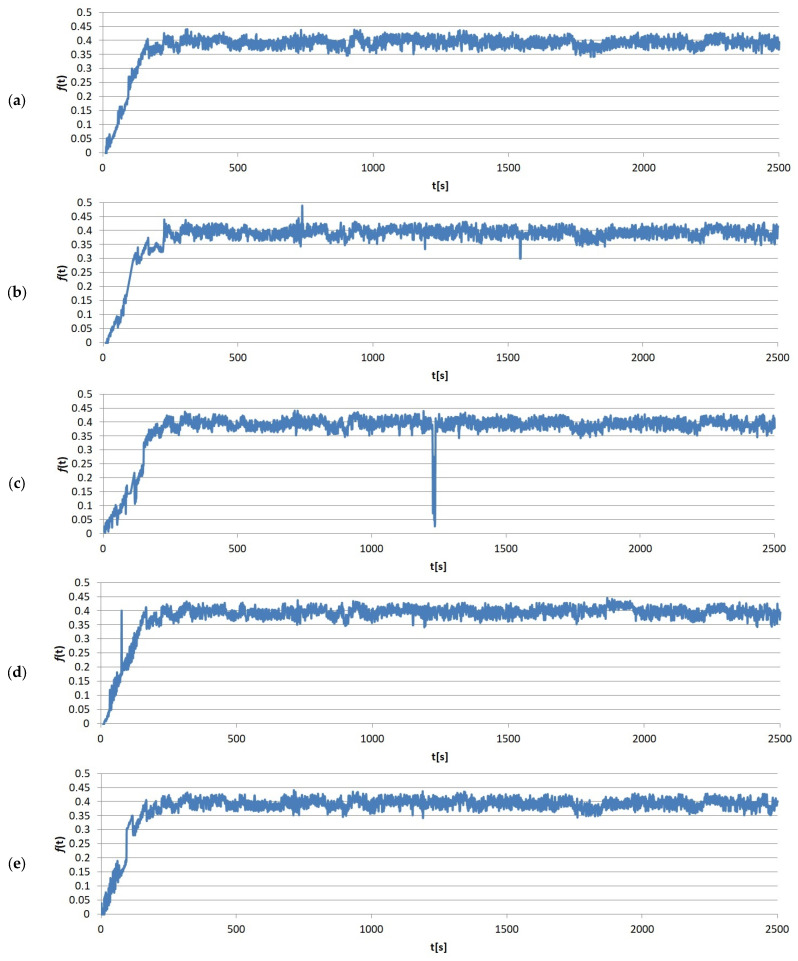
Example results graphs of the coefficient of friction of sample no. 10 from group S4: (**a**) run 1; (**b**) run 2; (**c**) run 3; (**d**) run 4; (**e**) run 5.

The immediate results of the friction tests were the friction force values measured over time. One of the sample measurements recorded by the T-20 stand, divided by load, is shown in Figure 5, Figure 6, Figure 7 and Figure 8. It clearly shows that the entire friction process during a single test can be divided into two periods: (1) running-in, where the geometric adjustment of the friction node takes place; (2) proper test, where proper contact occurs, allowing the actual value of COF between the tested materials to be obtained.

When analyzing the results, the entire friction period was discarded. Only the specific friction period was taken into account. The arithmetic mean was calculated from the recorded curves, each of which had approximately 2.2 × 10^3^ points. The Amontons–Coulomb friction law [28] was then applied:

(3) $$f_{ij}=\frac{{\bar{F}}_{ij}}{L},$$

where: *f*—coefficient of friction *i* run of *j* series of samples (where *i* = 1…5, *j* = 1…3), *F*—calculated average friction force; *L*—load. The values of friction coefficients were determined. The next step to obtain information on the discrepancies in the results was to determine standard deviations:

(4) $$S_{d}=\sqrt{\frac{\sum_{i=1}^{3}{\left(f_{j}-{\bar{f}}_{j}\right)}^{2}}{2}},$$

where: *f_j_*—average COF value of a single sample. The results are compiled in Table 4.

**Figure 9:**
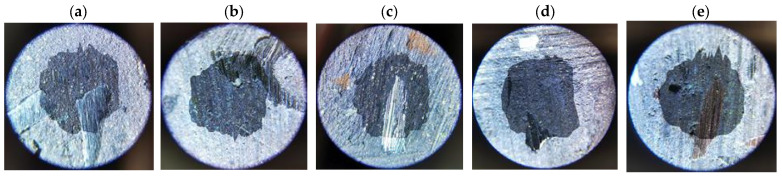
Example craters on sample no. 1 from group S1: (**a**) run 1; (**b**) run 2; (**c**) run 3; (**d**) run 4; (**e**) run 5.

**Figure 10:**
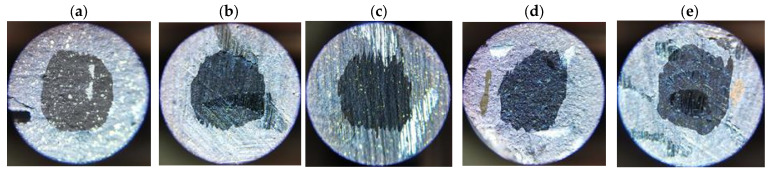
Example craters on sample no. 4 from group S2: (**a**) run 1; (**b**) run 2; (**c**) run 3; (**d**) run 4; (**e**) run 5.

**Figure 11:**
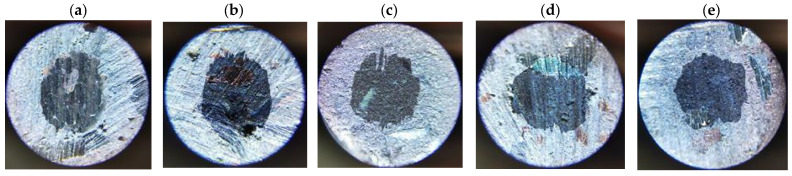
Example craters on sample no. 7 from group S3: (**a**) run 1; (**b**) run 2; (**c**) run 3; (**d**) run 4; (**e**) run 5.

**Figure 12:**
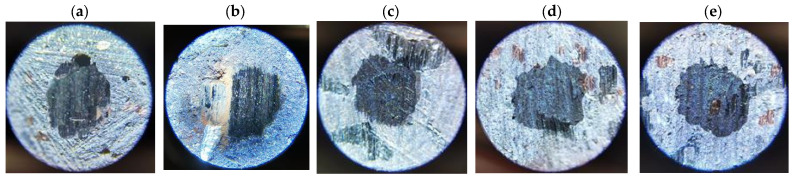
Example craters on sample no. 10 from group S4: (**a**) run 1; (**b**) run 2; (**c**) run 3; (**d**) run 4; (**e**) run 5.

Determining the values of the abrasive wear rate coefficients required additional measurements of the sizes of the craters formed in the samples as a result of friction (examples are given in Figure 9, Figure 10, Figure 11 and Figure 12). The measurement results and *k_c_* values determined using (2) are summarized in Table 5.

## 4. Discussion

**Figure 13:**
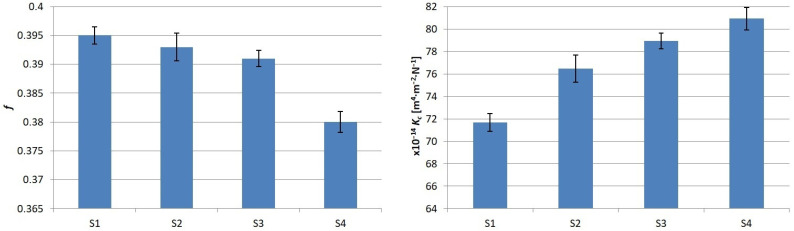
Results obtained during laboratory tests.

A graphical summary of the results obtained is shown in Figure 13. It can be clearly seen that after the introduction of flax fibers into the composite, there is a significant decrease in the value of the coefficient of friction with a simultaneous increase in the coefficient of abrasive wear rate.

The value of the friction coefficient directly affects the braking distance; therefore, from this point of view, the composite friction material intended for brakes should have a high friction coefficient [29,30]. On the other hand, a higher coefficient of friction causes an increase in temperature in the friction node and may cause accelerated wear of the working elements of the brakes [31,32]. Higher temperatures, in turn, may contribute to the fading phenomenon, widely described in the literature [33,34].

The obtained results confirmed the results of other researchers. The tests obtained lower values of the friction coefficient when using plant-based reinforcement. However, the differences from the reference sample were not large, and the values themselves were more favorable than those obtained in other studies of this type [12,13,14,15].

A statistical analysis was carried out in order to accurately estimate the effect of the type of reinforcement. Since the influence of the factor (type of reinforcement) on the selected parameter (COF—coefficient of friction) was studied, the most advantageous method was the use of one-way analysis of variation (ANOVA) [35,36]. It was assumed that the calculations would be performed with the confidence level: α = 95%. The first step was to determine the degrees of freedom for the qualitative factor, random error, and total variation. The following equations were used:

(5) $$D_{fa}=a-1,$$

(6) $$D_{fe}=N-a,$$

(7) $$D_{ft}=N-1,$$

where: *a*—the number of objects in the entire experiment; *N*—the number of experimental units in the entire experiment. The correctness of the calculation is checked by satisfying the following equation:

(8) $$D_{fa}+D_{fe}=D_{ft}.$$

The equation proved to be true, so the next step was to calculate the sums of squares. Sums were also calculated for three factors: the qualitative factor, random error, and total variation. The necessary data were substituted into the following formulae:

(9) $${SS}_{a}=\sum_{i=1}^{a}n\left({\bar{x}}_{i}-\bar{x}\right),$$

(10) $${SS}_{e}=\sum_{i=1}^{a}\sum_{j=1}^{n_{i}}\left(x_{ij}-{\bar{x}}_{i}\right),$$

(11) $${SS}_{t}=\sum_{i=1}^{a}\sum_{j=1}^{n_{i}}\left(x_{ij}-\bar{x}\right).$$

where: *n*—number of repetitions; ${\bar{x}}_{i}$—object mean; $\bar{x}$—overall mean; *x*—value of a single measurement for sample no. And there must be a relation between *SS* values:

(12) $${SS}_{a}+{SS}_{e}={SS}_{t}.$$

The mean values for the qualitative factor and the random error took the following forms:

(13) $${MS}_{a}={SS}_{a}/D_{fa},$$

(14) $${MS}_{e}={SS}_{e}/D_{fe}.$$

The results are summarized in Table 6.

Determining the above values allowed us to finally calculate the *F-Fisher* function from the following equation:

(15) $$F_{f}=\frac{{MS}_{a}}{{MS}_{e}}.$$

The obtained values, at the *α* confidence level, were compared to the critical value read from statistical tables:

(16) $${F_{S1\ldots S4}>F}_{crit}=2.247.$$

They differ very little. However, thanks to the difference of 0.03, with the assumed confidence level *α* = 95%, it should be concluded that the hypothesis stating the lack of influence of reinforcement on the value of the friction coefficient, i.e.,

(17) $$H:\;f_{S1}=f_{S2}=f_{S3}=f_{S4},$$

should be rejected. This is also confirmed by the determined *p*-values. Then, the homogeneity of samples in each group was determined. For this purpose, the Levene test was used [37] (Table 7):

(18) $$F_{Lev}=\frac{\sum_{i=1}^{a}n_{i}{\left({\bar{x}}_{i}-\bar{x}\right)}^{2}/(a-1)}{\sum_{i=1}^{a}\sum_{j=1}^{n_{i}}n_{i}{\left({\bar{x}}_{ij}-\bar{x}\;\right)}^{2}/\sum_{i=1}^{a}\left(n_{i}-1\right).}$$

The results show that the samples of groups S1, S2, and S4 can be considered homogeneous at a confidence level of *p* = 5%. However, the S3 group of specimens shows small group differences. This means that individual samples show differences in their tribological properties. These differences are most likely due to a manufacturing error wherein one of the S3 samples was slightly different from the others. It could also be due to the fact that despite intensive mixing of the semi-finished product, the components were arranged in a certain characteristic way; e.g., several hard steel particles were close together.

It was also shown that there is a statistically significant effect of the reinforcement method on the friction coefficient values. Importantly, the value that allows such a statement to be made is extremely close to the critical value. A slight increase in the significance level would allow hypothesis (17) to be accepted.

From the graphical interpretation of the results, it can be seen that the greatest decrease in COF value was observed after the complete elimination of aramid, although numerically, this value is negligible in practical use (only 0.1).

The graph of the abrasive wear intensity coefficient shows the opposite trend: as the aramid content decreases, the wear of the samples becomes more intense.

## 5. Conclusions

This manuscript presents the results of tribological tests on a composite friction material in which the conventional aramid reinforcement has been replaced by a more ecological material in terms of both production, operation, and disposal—flax fibers. No such use of flax fibers was found in the analyzed scientific works. This is even more important because these fibers are widely available and, above all, they are ecological. By using such a solution in motor vehicles, it would be possible to limit the emission of harmful compounds into the environment, as well as to reduce the consumption of aggressive chemicals currently used in the production of friction materials or their components.

A ball-cratering method was used for the research. The tests were carried out on a “ball-cratering” test rig with the factory designation T-20. As a result of this research, the following conclusions can be drawn:

- There was a negligible effect of changing the reinforcement material, both in proportions (S2 and S3) and in the complete replacement of aramid by flax (S4);
- There was small effect, around 10%, of the change in reinforcement on the abrasive wear rate, which increased when flax was used as reinforcement.

From the tribological tests carried out, it can be concluded that it is possible to replace aramid with flax fibers in composite friction materials. This is particularly true for light vehicles traveling at low or medium speeds.

The tests were carried out at low loads and relative speeds. For this reason, it can be assumed that the test took place at a constant temperature. Studies have repeatedly shown [38] that temperature has a significant impact on the value of the friction coefficient. Determining the temperature sensitivity of the friction coefficient of the proposed materials will be the subject of further research.

## Figures and Tables

**Table 2:** Orthogonal table listing the input parameters of the preliminary tests.

Preliminary Test No	Load [N]	Distance [m]	Rotation Speed [RPM]
1	2	50	38
2	2	100	80
3	2	150	150
4	4	50	80
5	4	100	150
6	4	150	38
7	6	50	38
8	6	100	150
9	6	150	80

**Table 3:** Preliminary tests results.

Preliminary Test No	Average Friction Force Value [N]:				
	1	2	3	4	5
1	0.37	0.38	0.36	0.37	0.42
2	0.54	0.55	0.58	0.51	0.54
3	1.01	0.96	0.96	1.13	1.04
4	1.09	1.14	1.14	1.25	1.28
5	1.51	1.49	1.43	1.39	1.47
6	1.14	1.46	1.17	1.13	1.37
7	1.12	1.05	0.82	1.04	1.14
8	0.76	1.09	1.07	0.82	0.81
9	1.91	1.80	1.81	1.83	1.90

**Table 4:** The results of the average COF in individual tests.

Group No	Sample No	COF Value					Average	Standard Deviation
		Run No 1	Run No 2	Run No 3	Run No 4	Run No 5		
S1	1	0.42	0.40	0.37	0.38	0.39	0.396	±0.015
	2	0.39	0.39	0.42	0.41	0.38		
	3	0.39	0.39	0.39	0.41	0.38		
S2	1	0.41	0.38	0.41	0.39	0.43	0.393	±0.024
	2	0.41	0.41	0.37	0.38	0.42		
	3	0.35	0.37	0.42	0.35	0.38		
S3	1	0.40	0.38	0.41	0.41	0.41	0.391	±0.014
	2	0.39	0.39	0.40	0.39	0.36		
	3	0.37	0.39	0.41	0.38	0.38		
S4	1	0.39	0.38	0.39	0.37	0.38	0.379	±0.018
	2	0.38	0.38	0.40	0.35	0.39		
	3	0.36	0.42	0.38	0.38	0.36		

**Table 5:** Crater diameter measurements results and calculated abrasive wear rate values.

Group No	Sample No	Crater Diameters [mm]		Average Diameter [mm]	*k_c_* [m^4^·m^−2^·N^−1^]
		In the Direction of Friction	Perpendicular to the Direction of Friction		
S1	1	1.95	1.78	1.842	71.68 × 10^−14^
	2	1.84	1.88		
	3	1.81	1.79		
S2	1	1.91	1.82	1.871	76.46 × 10^−14^
	2	1.94	1.91		
	3	1.8	1.85		
S3	1	1.79	1.88	1.886	78.94 × 10^−14^
	2	1.82	1.91		
	3	1.93	1.99		
S4	1	1.87	1.94	1.898	80.92 × 10^−14^
	2	1.91	1.85		
	3	1.96	1.86		

**Table 6:** ANOVA analysis calculations—single-dimension results.

Source of Variation	*D_f_*	*SS*	*MS*	*F_f_*	*p*
qualitative factor	3	2.32 × 10^−3^	7.73 × 10^−3^	2.25	93.22 × 10^−3^
random error	54	18.56 × 10^−3^	0.34 × 10^−4^		
total	57	20.88 × 10^−3^			

**Table 7:** Levene test results.

	Samples Group			
	S1	S2	S3	S4
*F_Lev_*	2.007	3.409	5.352	0.865
*p*	0.177	0.052	0.014	0.865

## Data Availability

Data are contained within the article.
